# The process and motivations of individual values internalization: a qualitative study

**DOI:** 10.1186/s40359-025-03424-2

**Published:** 2025-09-26

**Authors:** Lanting Wu, Youguo Chen, Xiting Huang

**Affiliations:** 1 Faculty of Psychology, Southwest University, Chongqing, 400715 China; 2 Research Center for Psychology and Social Development, Southwest University, Chongqing, 400715 China

**Keywords:** Qualitative research, Values internalization, Process, Motivations, Characteristics

## Abstract

Values influence various aspects of an individual’s life. Current theories on values internalization primarily consider from the observer’s perspective, lacking information from the subjective perspective of the individuals internalizing these values. To understand the process of values internalization in real-life contexts, this study employed qualitative interviews, conducting semi-structured interviews with 20 interviewees. The interview data were coded using thematic analysis. The results indicate that: (a) the process of values internalization includes four stages: ignoring-resisting stage, understanding stage, attempting to practice stage, and integration stage. (b) These stages exhibit distinct characteristics in terms of cognition, behavior, motivation, emotion, self-relevance, importance, and priority. And (c) the primary internal factors influencing values internalization are achieving positive outcomes and avoiding negative consequences, while major life events and peer behavior are the main external factors. This study reveals the process and characteristics of values internalization from the individual perspective, expanding and supplementing the observer’s perspective on the theory and research of values internalization, and providing a more comprehensive theoretical foundation for measuring.

## Introduction

Values are deeply embedded in all aspects of life, shaping individuals’ decisions about careers, education, relationships, residence, consumption, health, and more. Values are generally defined as concepts or beliefs about desirable end states or behaviors that transcend specific situations, guide the selection or evaluation of behavior and events, and are ordered by relative importance [1]. Understood in this way, values differ from attitudes in their level of abstraction, generality, and their hierarchical structure [1]. While the content of specific values may vary across individuals and cultures, Schwartz’s model proposes a universal structure of ten basic value types that are recognized across cultures: self-direction, stimulation, hedonism, achievement, power, security, conformity, tradition, benevolence, and universalism [1–3]. Cross-cultural research has demonstrated that these ten values are universally recognized and have near-universal meaning across culturally diverse groups [4, 5].

Importantly, values are not innate; individuals are not born with a predefined set of values. Rather, values are gradually formed and internalized through social interactions and life experiences, particularly under the influence of cultural norms, family socialization, and broader societal expectations [6, 7]. Through this developmental process, external value systems are progressively integrated into one’s personal value structure, forming a relatively stable framework that guides behavior and self-regulation. The concept of “internalization” has been widely used to describe this process of adopting external or societal values as one’s own [8]. Values internalization refers to the psychological process by which individuals come to accept and embody external values, such that socially desirable behaviors are driven not by external enforcement, but by internal motivation [6]. Understanding how values become internalized is thus essential for uncovering how individuals form, maintain, and act upon their value systems.

Internalization has been discussed in various theories, notably by Piaget [9] and Vygotsky [10]. Piaget, through structured observations and experiments with children solving moral and logical problems, views internalization as the process of conceptualization [11], where individuals respond to external stimuli by assimilating and accommodating them into their cognition, maintaining cognitive balance through self-regulation and equilibrium [9]. However, Piaget’s focus is largely structural and developmental, emphasizing cognitive mechanisms over motivational or emotional dimensions, and relying on behavioral inferences rather than the individual’s subjective meaning-making during internalization. Vygotsky, from a sociocultural perspective, based on his observational studies and theoretical analysis of language development and learning in children, divided the internalization process into four stages: from primitive natural behavior to internalized, self-regulated activity mediated by cultural signs and tools [10]. His theory foregrounds the social origins of internalization, notably through concepts like the “zone of proximal development”. However, while Vygotsky enriched the understanding of how social interaction facilitates value acquisition, his framework does not engage with how individuals personally interpret or emotionally relate to these values once internalized.

In addition to Piaget and Vygotsky, Kelman [12] approached internalization from a motivational and social influence perspective, particularly in the context of attitude and behavior change. Drawing from extreme cases like ideological transformations of “believers”, Kelman [12] identified three processes of social influence: compliance (external motivation), identification (motivated by social relationships), and internalization (adoption of values as intrinsically meaningful). His work emphasizes that true internalization occurs only when external values are congruent with the individual’s value system. Nevertheless, Kelman’s framework does not detail the cognitive or emotional processes by which such congruence is achieved.

Similarly, Deci et al. [13] developed the self-determination theory (SDT) from a motivational perspective, based on experimental and longitudinal research, which distinguishes between two types of value internalization: introjection, where values are adopted due to external pressure, and integration, where values are fully assimilated and actions are autonomously motivated [13]. Although SDT distinguishes between different motivational qualities, it conceptualizes internalization primarily through observable behavioral outcomes, rather than exploring how individuals subjectively experience and navigate these transitions over time.

Additional models, such as Soviet psychologist Gal’perin’s stages theory of mental action [14, 15] and American psychologist Bloom’s taxonomy of affective development [16, 17], provide staged frameworks for how external knowledge or values are transformed into internal competencies or stable dispositions. While these models enrich the structural and pedagogical understanding of internalization, they too are primarily grounded in third-person observation and developmental sequencing, and rarely account for the lived, situated, and affective nature of values adoption.

Together, these theoretical perspectives provide a multifaceted foundation for understanding internalization, highlighting its developmental, social, and motivational dimensions. Common across these frameworks is the recognition that internalization involves a transformation from external input to internal structure, often through interaction, reflection, and meaning-making. While effective in understanding external behaviors, third-person approaches struggle to capture conscious experiences, which cannot be observed directly. Consciousness, being subjective, requires a first-person perspective [18]. In contrast to dominant third-person approaches, several phenomenological and narrative frameworks have sought to explore internalization from the actor’s first-person perspective. For instance, narrative identity research has examined individuals in late adolescence and young adulthood are likely to focus some of their identity development work on crystallizing the basic values and beliefs [19, 20]. Phenomenological studies have also investigated first-person experiences of moral or ideological transformation [21]. These approaches provide valuable insights into subjective experiences. However, much of this work has focused on specific contexts—such as religious conversion [22, 23], identity development [24], or intergenerational value transmission [25]—and has not mapped the general process and stages of individual values internalization across diverse values.

Building on these traditions, this study adopts an emic perspective, aiming to understand how individuals themselves perceive, make sense of, and internalize values within their sociocultural contexts [26, 27]. Participants were invited to narrate what they considered important and how these values evolved. This emic orientation allows for capturing lived, subjective experiences of value internalization, which are often overlooked in etic or third-person frameworks [28].

To align with this perspective, the study employs a qualitative research approach, which is particularly suited to exploring subjective processes in depth. This choice is based on three main considerations: (a) its inductive logic, (b) its ecological validity, and (c) its theoretical contribution. First, the logic of qualitative research is inductive reasoning. This means that through direct observation and linguistic descriptions in natural settings, researchers can articulate the thoughts, feelings, and behaviors of respondents. By continually refining the data, a clearer and more comprehensive understanding of certain psychological and behavioral phenomena can be achieved. This aligns with the logic of studying values internalization from the actor’s perspective. Second, qualitative research offers strong ecological validity. By immersing in people’s actual lives and empathetically understanding their psychology and behavior, researchers can gain a clearer and more thorough understanding of the occurrence and development of events through the narratives of the individuals themselves. Third, qualitative research contributes to theoretical development. Through in-depth understanding of individual cases, qualitative research aims to continuously develop and improve human cognitive structures, thereby providing new perspectives and questions for social science research, and allowing researchers to gain broader insights and deeper comprehension [29].

In line with these strengths, this study uses semi-structured interviews with adults of various ages and occupational backgrounds. By situating interviews within participants’ daily contexts and focusing on subjective meaning-making, the study aims to reveal the process of individual value internalization and to provide a theoretical basis for fostering the internalization of positive values in practice.

## Materials and methods

Using interviews as a data collection method within qualitative research allows for a deeper understanding of respondents’ thoughts, emotional responses, life experiences, and the underlying meanings of their behaviors [29]. This approach is particularly well-suited to the purpose of the present study, which is to explore the process of individual values internalization and its influencing factors by drawing on participants’ lived experiences and personal narratives. Although interviews rely on retrospective self-report, previous research has shown that adults are capable of meaningfully reflecting on significant life events and reconstructing narratives that reveal perceived developmental stages [30, 31]. To enhance the richness and reliability of retrospective accounts, we employed open-ended questions and follow-up probes to help participants recall concrete examples and contextual details. The interviews were conducted in a semi-structured format, where the interviewer maintained some control over the structure but also encouraged respondents to actively participate by introducing their own questions. The interview was guided by a general interview guide, with flexibility to adjust according to the specific dynamics of each interview.

The interviewer, a psychology doctoral student skilled in counseling techniques, conducted the interviews. Throughout the process, the interviewer maintained an attitude of respect and sincerity, effectively employing techniques such as active listening, positive regard, and clarification. Most importantly, the interviewer focused solely on uncovering facts while maintaining a value-neutral stance, refraining from evaluating the information provided by respondents. This approach is designed to avoid influencing respondents’ perspectives while also encouraging them to engage in self-reflection, thereby obtaining firsthand research data.

### Interview guide

To ensure conceptual clarity during the interviews, participants were first introduced to the definition of values based on Schwartz’s theory. Values were described as trans-situational guiding principles that influence the selection and evaluation of behavior and events, and are ordered by relative importance. Although participants were not directly asked to list or define their values, the interview guide included open-ended questions such as “What is most important to you in life?” and “What principles matter most to you when making decisions?” These questions encouraged participants to reflect on their core beliefs. They were further invited to share how these values were formed, influenced, and possibly changed over time. This interview design helped ensure that the data collected focused on personally meaningful and enduring values rather than on temporary preferences or context-specific attitudes.

Based on a review of the literature and alignment with the study’s research objectives, a preliminary interview guide was drafted. This draft was then revised, supplemented, and finalized by two doctoral students with practical interview experience, resulting in a broadly structured interview guide. The interview guide is organized as follows:

- Demographic information: including age, gender, etc.
- Questions to encourage expression: understanding the respondent’s daily life or work situation.
- Core research questions: exploring the process, manifestations, causes of values change, and experiences when values were violated.
- Concluding questions: gathering respondents’ additional thoughts, feelings, and suggestions regarding the interview, and thanking them for their participation.

The specific questions for the core research section are as follows:

- What is most important in life to you? What are the key principles you live by? What principles matter most to you when making decisions?
- When did you start having these thoughts? How are these thoughts reflected in your life?
- What were your previous thoughts on this? Why did your thoughts change?
- Did your life change after your thoughts changed?
- What was the initial change like? Have there been further changes since then?

### Participants

In this study, a combined sampling strategy was employed, including stratified purposive sampling, intensity sampling, and convenience sampling, to select suitable participants. Although 21 participants were initially interviewed, one was excluded due to poor data quality, resulting in a final sample of 20. Thematic saturation was reached around the 17th interview, with no new themes emerging in the subsequent ones, in line with established qualitative research guidelines [32, 33]. The participants ranged in age from 19 to 58 years (*M* = 29.20, *SD* = 11.08). In terms of educational background, high school education accounted for 5%, junior college education accounted for 30%, university education accounted for 40%, and master’s degree and above accounted for 25%. The sample consisted of 7 males and 13 females, with occupations including students (40%), company employees (25%), a middle school teacher (5%), university teachers (10%), a housewife (5%), and retirees (15%).

### Procedure and data collection

When recruiting interviewees, they were informed about the interview content, time, duration, location, format, and guiding principles. The interviews proceeded only after reaching a mutual agreement. Before each interview, the purpose and format of the research were explained again, along with the principles of voluntariness, confidentiality, and the right to withdraw at any time. After obtaining the interviewee’s consent, an informed consent form was signed, and the interview was recorded. The interview content was guided by an outline, with the primary focus on the interviewee’s values. After each interview, the quality and information were summarized and reflected upon, allowing for adjustments to the interview outline in preparation for the next session. This study was conducted and reported in an ethically appropriate manner and received ethical approval from the ethics committee of the researcher’s institution.

### Data analysis: thematic analysis

The in-depth interview recordings (S1-S20) were transcribed into electronic text, capturing not only the spoken words but also the interviewees’ changes in facial expressions, tone of voice, and pauses in thought. The final transcripts amounted to a total of 17 h, 43 min, and 24 s of recording time, resulting in over 236,000 words.

Thematic analysis was employed to identify and refine core themes from the qualitative data [34]. We followed a three-stage coding process, assisted by the qualitative data analysis software QSR NVivo 11.0, created initial codes directly from participants’ words, grouped them into categories, and then synthesized them into themes [35, 36]. NVivo features such as node creation, hierarchical coding, coding comparison queries, matrix coding, and visual model mapping were systematically used to support coding management, comparison, and theme development.

The analysis process consisted of the following stages:

- The primary themes: Coding was performed on the 20 interview transcripts. The coding captured the meaning expressed by the interviewees, with an effort to use the exact words spoken by the interviewees as codes. The coding of primary themes is shown in Table 1. This stage resulted in 816 reference points and 86 codes. During this phase, NVivo’s node creation and annotation features were used to record initial ideas and link codes to raw excerpts.
- The secondary themes: The results from the coding of primary themes stage were further merged, extracted, and named through comparisons of similarities and differences, both horizontally and vertically. The naming principle was to use terms close to the interviewees’ original descriptions, expressing common concepts without losing their meaning. NVivo’s matrix coding query function helped visualize code co-occurrence and supported the merging of closely related codes. This stage yielded 23 higher-order thematic codes.
- The core themes: In the final stage, the categories identified in the previous phase were synthesized into 7 core themes. This process involved iterative reviews of thematic connections, conceptual overlaps, and representative quotes to ensure thematic saturation and coherence. NVivo’s visual model builder was used to map relationships among codes and themes, facilitating theory-driven integration.

**Table 1:** The coding of primary themes (Excerpt)

Original text of S1 about changes in health values	Primary themes
**Interviewer**: Has there been any change after your shift in mindset? **interviewee (S1)**: Yes, definitely. For example, a noticeable change in my daily life is that, first, I started drinking more water than before, and now I do it almost subconsciously. For instance, when I arrive at the office, the first thing I might do is decide what kind of water to drink that day, but I always make sure to fill my cup and drink it. **The first change is that I now drink water consciously and have made it a habit (a).** I also practice yoga and try to adjust my work schedule so that I can participate in physical exercise more frequently each week. **I no longer cancel exercise because of work; instead**,** I shift my work schedule to make room for it (b).** Now, I actively consider these adjustments. Additionally, in my daily life, I often remind myself after certain activities that they may not be good for my body, and I react accordingly. **For example**,** when my shoulders start to hurt from prolonged desk work**,** I remind myself to do some stretches (c). Although it’s not constant**,** it does occur more frequently now (d).**	(a) Develop habitual behaviors; (b) Values are more important than other things; (c) Adjust behavior after violating values; (d) Increased frequency of practicing values

To enhance the rigor and credibility of the qualitative data analysis, multiple validation strategies were employed. First, the researcher engaged in continuous reflexivity, maintaining analytic memos to document decision-making processes and reduce potential bias during coding [37]. Subsequently, peer debriefing was conducted throughout the coding and analysis process [38, 39]. The researcher held multiple rounds of discussion and critical review sessions with a psychology associate professor and two doctoral students in the field, leading to refinements of the thematic structure. Finally, member checking [40] was carried out with 10 participants, who were invited to comment on whether the coded summaries and thematic interpretations reflected their intended meanings, and any suggested adjustments were incorporated.

## Results

The values involved by the interviewees are shown in Table 2. Using thematic analysis, the 20 transcripts were coded, ultimately identifying 7 core themes related to the internalization of values. These include 6 core themes representing the stages of the values internalization process: ignoring values, resisting values, understanding values, attempting to practice values, transforming values, and stabilizing values. Analysis of the results revealed certain inherent logical relationships among these 6 core themes, leading to the further categorization of the values internalization process into four stages: ignoring-resisting stage; understanding stage; attempting to practice stage; integration stage. In addition to these 6 core themes representing the stages of values internalization, another core theme, Motivations for Values Internalization, was identified, which includes both intrinsic and extrinsic factors. The specifics are as follows:

**Table 2:** Values mentioned by interviewees

Interviewees	The values mentioned by the interviewees	Interviewees	The values mentioned by the interviewees
S1	Happiness; Physical health	S11	Physical health; exercise; Friendship、Cautious about speech
S2	Reduce internal psychological conflict; Family	S12	Finding a good job; Family; Being kind
S3	Family; Living environment; Sincerity and authenticity	S13	Physical health; Positive life attitude; Helping other; Sincerity and authenticity
S4	Family; Sincerity and authenticity; Maintaining work boundaries	S14	Happiness; Expressing needs; Friendship; Adhering to moral standards; Religious beliefs
S5	Effort will be rewarded; Happiness; The sense of life experience; Providing for parents	S15	Physical health; Family; Sincerity and authenticity; Being kind
S6	Family; Be flexible and adaptable in handling matters	S16	Career orientation; Family; Views on childbearing; Family; Helping other
S7	Physical health; Physical health; Personal freedom; Self-acceptance; Do not impose on others what you do not desire	S17	Physical health
S8	Learning; Friendship; Family; Leave room for future encounters	S18	Physical health; Sincerity and authenticity; Helping other; Friendship
S9	Family; Striving for a better future and ideals for the people	S19	Physical health; Do no harm to others
S10	Money; Self-discipline; Learning; Not speaking ill of others	S20	Physical health; Do no harm to others; Helping other

### Stages of values internalization

The stages of value internalization can be divided into four major stages, as detailed in Table 3. The first stage is the ignoring-resisting one, which includes two core themes: ignoring values and resisting values. In this stage, individuals have not internalized the values. Ignoring values refers to when individuals are not yet aware of the importance of the values and do not hold those values. Resisting values refers to when individuals reject the values and hold opposing beliefs. The main manifestations of ignoring values include: (1) Low relevance of values to life. Values are rarely involved in an individual’s life, are not considered in daily living, or seem distant from one’s life. For example, “*It’s a correct statement*,* but it’s not that important to my personal life or doesn’t really relate to me personally (S1)*.” (2) Low importance of values. For the individual, the importance of values is perceived as lower than that of other matters, such as self, future, friends, happiness, work, or study. Additionally, the individual may not value or be aware of the importance of values. For example, “I *might spend more time on other things rather than focusing on this aspect (S3)*”; “*I used to think it didn’t matter*,* that there were no taboos (S19).*” Resisting values is manifested in the following ways: (1) Behavior inconsistent with values. The respondents don’t practice the values, adhere to other values, or behave in ways that contradict the values. For example, S16 discussed their work philosophy when he was younger: “*At that time*,* I was still very interest-driven because I found this type of work very interesting.*” (2) Negative emotions towards values. When the respondents practice the values or encounter information related to the values, they may experience negative emotions such as fear, anxiety, or resistance. For instance, S10 mentioned feeling rebellious when her family urged her to be self-disciplined: “*Even after I got to college*,* my parents would still scold me for playing games*,* and then I’d feel rebellious.*” (3) Rejecting values information. The respondents’ thoughts may differ from the values, or they may not believe in or understand the values. For example, S19 reflected on hearing advice from elders when she was younger: “*Oh*,* I just didn’t understand it*,* and I didn’t take it to heart.*”

The second stage is the understanding stage. During this phase, individuals rethink their values, eliminate resistance to values, or recognize their importance, and begin to understand the values. This stage includes one core theme: understanding values. In the understanding stage, respondents exhibit the following characteristics: (a) Low priority of values. Although they start to recognize the importance of values, they do not particularly emphasize them, and values are given a low priority. For instance, happiness may be considered more important than health (S11), or work may be considered more important than health (S15). (b) Initial understanding of values (superficial understanding). Their understanding of values is unclear, partial, or superficial. They may see values as mere principles. For example, S6 reflected on his earlier view of family harmony: “*It was simply because of arguments that people brought up this statement.*” (c) Acceptance of values but difficulty in adhering to them in behavior. At this stage, respondents begin to change their views and accept the values. However, these values do not guide their behavior. Some respondents do not adjust their actions based on the values, while others forget to follow the values or acknowledge their importance, but fail to act accordingly. For example, “*I think money is a very important conceptually*,* but in practice*,* I didn’t have the intention to make money*,* and it was also beyond my ability (S10).*”

The third stage is the attempting to practice stage. After individuals’ understanding of values changes, they attempt to practice these values, driven by external motivations, while continuing to accumulate their understanding of the values. This stage includes one core theme: attempting to practice values. During this stage, (a) External motivation drives individuals’ behaviors. This includes influences from family education, school education, interpersonal relationships, and benefit-driven motives. For example, “*Anyway*,* I found it really difficult*,* very difficult indeed*,* but my family kept urging and supervising me (S19)*,” and “*At first*,* she might have led us*,* and then I just followed along (S3)*.” (b) Difficulty in practicing values. There is an attempt to practice values, but it may be infrequent, with limited time devoted to it, difficulty in sustaining behavioral changes, and challenges in adapting to new behaviors. For instance, “There wasn’t much time for exercise (S7),” and when S18 started focusing on health, she said, “Sometimes I couldn’t resist (eating junk food). I’d crave it, because after all, some junk food tastes really good.” (c) Emotional reactions/fear of living out values. Individuals may feel anxious, nervous, or fearful of failure during this stage. For example, “I was a bit nervous, a bit scared, so I took it very seriously (S5).”

The fourth stage is the integration stage. After going through the initial understanding of values and the trial implementation, individuals’ internalization of values deepens, leading them into the integration stage. During this stage, individuals abandon their previous beliefs, actively practice and embrace the values, and eventually, these values become stable and guide their lives. The integration stage includes two core themes: transforming values, and stabilizing values. In the process of value transformation, individuals demonstrate the following: (a) Increased proactivity in following values. Individuals become more consciously aware of values and actively consider them in their daily lives. For instance, as S1 mentioned, “Now I feel the need to consciously focus on this aspect.” Additionally, compared to before, they pay more attention to values and related information in their lives. For example, S16 expressed increased attention to acts of kindness, “*So if anything similar happens*,* I will pay close attention and be deeply moved.*” (b) Efforts to practice values. As proactivity in following values increases, so does the effort individuals put into practicing these values. They adjust their behavior according to values, strive to adhere to them, and increase the frequency and level of commitment to practicing values. (c) Value endorsement. This is a cognitive characteristic where individuals develop a deeper understanding and acceptance of values. For example, S2 discussed family relationships, saying, “*Compared to before*,* I now believe that maintaining this kind of relationship among family members is essential.*” When values become stable, individuals exhibit the following characteristics: (a) Values are related to self. Values become deeply embedded in the individual’s life, influencing daily actions. For example, S1 mentioned her current view on the importance of health, saying, “*It has become deeply ingrained in my daily life.*” (b) High importance and priority. Individuals prioritize values, considering them foundational and crucial. These values become more important than they were before and more significant than other values. For instance, S15 highlighted the importance of health, stating, “*So I believe that health is the most important. If you are not healthy*,* nothing else matters.*” (c) Reactions to violating values. When individuals hold values stably, they reflect and adjust their behavior if they violate their values, working to avoid future violations. Emotionally, they may experience fear or discomfort, and they might become angry when others violate these values. (d) Expression of internal motivation. Individuals are driven by internal motivation to practice their values. They proactively seek out knowledge related to their values, follow them voluntarily, and spread these values to others. For example, S11 mentioned, *“After that*,* I started educating people around me on how to take care of their necks.*” (e) Enjoyment of a value-aligned life. After values have changed, individuals maintain a positive attitude toward aspects of life corresponding to their values. For example, S1 mentioned that after she began to prioritize health, she perceived reminders from close others as expressions of care: “*Now*,* when my parents or people close to me tell me to pay attention to my health*,* I feel that this behavior reflects their love and concern.*” (f) Behavior guided by values. As the degree of values internalization reaches its peak and internal motivation strengthens, there is a high consistency between an individual’s behavior and their values. They habitually engage in value-aligned actions, and their sense of self-efficacy in these actions increases. For instance, S19, reflecting on the later stages of practicing health-related values, shared: “*I started eating less junk food*,* using less oil and salt*,* and gradually*,* I got used to it.*” (g) adhering to values. Cognitively, the individual’s thoughts align with their values, and they remain steadfast in their beliefs, even in the face of external persuasion or temptation. As S6 noted when encountering perspectives that conflicted with her values: “*I won’t change my thinking just because of others’ comments.*”

**Table 3:** Primary themes, secondary themes, core themes and stages of the values internalization process

Stages	Core themes	Secondary themes	Primary themes	Reference
First stage: the ignoring-resisting stage	Ignoring values	Low relevance of values to life	Values are not considered in daily life; Daily life seldom involves values; Values are distant from life	11
		Low importance of values	Other things are more important; Unaware of the importance of values; Make little of values	38
	Resisting values	Behavior inconsistent with values	Behavior contradicts values; Does not practice the value; Adheres to different values	46
		Negative emotions towards values	Trepidation; Fear; restrained; Anxiety; Resistance; Dismissive attitude; Sense of pressure	11
		Rejecting value information	Beliefs differ from values; Does not trust the values; Does not understand the values	38
Second stage: the understanding stage.	Understanding values	Initial understanding of values (superficial understanding)	A superficial understanding of values; Perceiving values merely as principles; An unclear understanding of values	18
		Low priority of values	Values are important but are given a low priority	3
		Acceptance of values but difficulty in adhering to them in behavior	Forgetting to adhere to values; Values are considered important but not practiced; Failing to adjust behavior according to values	11
Third stage: attempting to practice stage	Attempting to practice values	External motivation drives individuals’ behaviors	School education; Interpersonal relationships; Family upbringing; Driven by interest	20
		Difficulty in practicing values	Limited time for practice; Infrequent practice; Difficulty adapting to behavioral changes; Difficulty maintaining behavioral changes	22
		Emotional reactions/fear of living out values	Nervousness; Fear of failure; Anxious or uneasy	5
Fourth stage: integration stage	Transforming values	Increased proactivity in following values	Consciously think of values; Actively consider values in daily life; Pay more attention to values in daily life	42
		Efforts to practice values	Strive to adhere to values; Increased frequency of practicing values; Practice values more committed; Adjust behavior according to values	55
		Value endorsement	Accept values; A deeper understanding of values	21
	Stabilizing values	Values are related to self	Values influence all aspects of life; Values have changed life	3
		High Importance and Priority	Prioritize values; Values are an important consideration; Values are the foundation of other things else; Values are more important than before; Values are more important than other things	65
		Expression of Internal Motivation	Actively seek knowledge related to values; Actively follow values; Actively promote values	32
		Enjoyment of a value-aligned life	A more positive mindset after the change in values; Following values generates positive emotions; Understanding the values conveyed by others	24
		Behavior guided by values	Develop habitual behaviors; Competent, maintain practice values; Put values into practice	82
		adhering to values	Ideas are aligned with values; Adhere to values despite external temptations	65
		Reactions to Violating Values	Adjust behavior after violating values; Discontent with others’ violation of values; Engage in compensatory behavior after violating values; Experience negative emotions after violating values; Reflect on actions after violating values; Avoid violating values in the future	33
**Total reference**:				645

### Motivations for the internalization of values

The motivations for the internalization of values refer to the various factors that influence an individual’s process of internalizing values, which can be categorized into internal and external factors, as detailed in Table 4. Internal factors primarily relate to the individual’s own reasons. This research identified eight internal factors influencing values internalization, with the most prominent being (a) the desire to achieve positive outcomes and (b) avoiding negative consequences. These findings suggest that individuals place great importance on the outcomes of practicing or not practicing certain values. For example, S19 mentioned a change in her attitude toward health, “*As it was immediately life-threatening*,* I had no choice but to change.*” Similarly, S11 discussed changes in his perspective on exercise: “Exercising makes me happier, and my body becomes healthier.” (c) The pursuit of achievement is the third most significant internal factor. Individuals are motivated to internalize values to gain achievements, such as surpassing others (S10) or achieving good results (S5), or from the sense of accomplishment (S1) and satisfaction (S10) experienced during the process of practicing values. Emotionally, individuals tend to focus on (d) avoiding negative emotional experiences and (e) seeking positive ones. For instance, S7 described her struggle with self-acceptance: “I felt so miserable constantly reflecting on this… eventually, I just gave up.” On the other hand, S19 shared her feelings after attempting to change her lifestyle: “If the test results are good this month, and the doctor praises me, ‘saying you’ve done well’, it feels great, and I feel encouraged to keep going.” In addition to the internal factors mentioned above, (f) as individuals grow older, (g) improve their cognitive abilities, and (h) experience role transitions (such as moving from being a student to becoming a counselor, as in the case of S4), their understanding and perception of values may also change.

External factors influencing values internalization can be divided into eight categories. (a) Experiencing significant life events is the most frequently mentioned external factor. Major events such as the COVID-19 pandemic (S2), the death of a loved one (S11, S15), or failure in the college entrance examination (S5) can disrupt an individual’s existing values, prompting reflection and values change. As S13 reflected, “*Having had three near-death experiences*,* these significant life events have instilled in me a strong will to survive*,* no matter what difficulties I face.*” (b) Peer behavior is the second most common external factor, as the actions of peers (e.g., friends, classmates) can influence changes in an individual’s beliefs and practices. For example, S8 noted how his classmates’ persistence in studying despite setbacks changed his views on learning: “*One classmate was held back a year but still insisted on studying.*” (c) Maintaining relationships with others ranks as the third most common external factor. S3 mentioned that she began to pay attention to their living environment because she needed to consider others’ feelings. (d) Also tied for third in frequency is a change in living or learning environments. For instance, S14 highlighted how environmental changes provided opportunities for personal transformation: “*I think the change in environment was a major factor. The people I met at this school were all new to me*,* so I had a chance to start fresh.*” Similarly, S16 pointed out that increased environmental unpredictability led to changes in his beliefs: “*The environment became unpredictable*,* and unexpected situations became more frequent.*” (e) School education and supervision, as well as (f) family education and supervision, are ranked fourth. S16 mentioned how school education fostered a spirit of helping others: “*When I was young*,* we had activities like ‘Learning from Lei Feng Day*,*’ and moral education classes would instill similar values.*” Additionally, S7 discussed the role of family supervision in developing health-conscious habits: “My parents would monitor my sleeping and waking times.” (g) Another external factor is the approval of others, which also influences values internalization. When others offer praise and affirmation, respondents are more inclined to internalize those values. As S15 recalled, “*Since I was little*,* I often heard others praise my grandparents for doing good deeds. Growing up in such an environment*,* I felt proud whenever I did something good.*” (h) Media influence is another significant factor, as illustrated by S16, who mentioned that “*watching the ‘Touching China’ program during middle school*,* which featured the story of teacher Guo Benyu*,* inspired my willingness to help others.*”

**Table 4:** Secondary, primary, and core themes of values internalization motivation

Core Themes	Primary Themes	Secondary Themes	Reference
Values internalization motivations	Internal motivations	Achieving positive outcomes	35
		Avoiding negative outcomes	31
		Pursuit of achievement	13
		Cognitive development	12
		Avoiding negative emotion	11
		Role transitions	6
		Experiencing positive emotion	5
		Growth with age	2
	External motivations	Significant life events	15
		Peer behavior	11
		Maintaining relationships with others	7
		Changes of environments	7
		School education and supervision	6
		Family education and supervision	6
		Social approval	2
		Media influence	2
		**Total reference**:	171

## Discussion

**Fig. 1:**
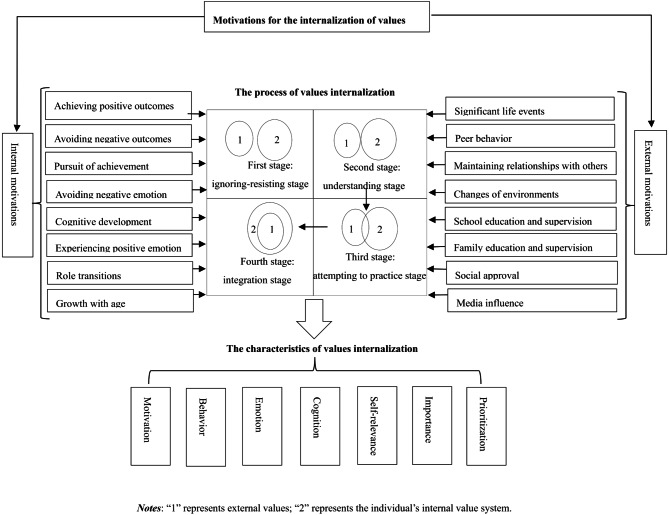
Structural model of the values internalization process and its antecedents and consequences

This study collected data on values changes from 20 participants using interviews, focusing on their perspectives. By systematically analyzing the interview data, the process of values internalization was categorized into four stages, and both internal and external factors influencing this process were identified. Based on the findings, a preliminary structural model of the values internalization process, including its antecedents and consequences, was developed (see Fig. 1). The following section discusses the results of this study.

### The process and characteristics of values internalization

This study divides the process of value internalization into four stages: ignoring-resisting stage, understanding stage, attempting to practice stage, and integration stage. These stages exhibit distinct characteristics across the following dimensions: (a) cognition, (b) behavior, (c) motivation, (d) emotion, (e) self-relevance, importance, and prioritization (see Table 5 for details). The specifics are as follows:

The ignoring-resistance stage refers to the phase in which an individual either remains unaware of the importance of certain values or resists adopting them. During this stage, individuals typically exhibit such as rejecting value-related information, acting in ways that contradict the values, harboring negative emotions toward the values, perceiving a low relevance of the values to their lives, and assigning low importance to these values. This stage has not been addressed in previous internalization theories, which primarily focus on the beginning of internalization or low levels of internalization (e.g., compliance in the theory of Kelman [12], or the lack of internalization in Deci et al. [13] self-determination theory). These theories overlook the state of individuals before the onset of internalization, where they may ignore or resist the values. Research by van Strien et al. [41] suggests that prior attitudes influence how individuals process conflicting information. Individuals tend to seek information that aligns with their existing attitudes, thus ignoring contradictory information. Given the limited capacity for information processing [42], selected information is easily amplified and remains in the “spotlight” of attention, while unselected information falls into the “dark side” of consciousness, outside the scope of attention [43]. Therefore, when value-related information is ignored, it struggles to enter the individual’s information processing system. Furthermore, when individuals believe that resisting external information is justified, they are more likely to resist external persuasion [44–46]. Thus, when individuals resist certain values, external information struggles to persuade them to adopt those values. Consequently, the ignoring-resistance stage is a critical phase in the process of values internalization that demands attention. Previous internalization theories, based on the perspective of external observers, have failed to explore the internal processes of individual values change, thereby neglecting the exploration of the ignoring-resistance stage. Only by addressing an individual’s neglect or resistance to certain values can the door to values internalization be opened, gradually deepening the internalization process until the values are fully integrated into the individual’s values system.

The understanding stage refers to the phase in which values begin to capture an individual’s attention, and the individual starts to develop an understanding of these values. At this stage, individuals typically exhibit the following characteristics: a preliminary and often superficial understanding of values, acceptance of the values but difficulty in adhering to them in practice, and low prioritization of these values. Vygotsky’s concept of the “naïve” level of internalization describes similar cognitive and behavioral traits observed when individuals first encounter external tools—they have yet to fully grasp their function or how to use them [10]. The key difference, as highlighted in this study, is that while individuals may cognitively accept the values, they struggle to align their behaviors with them. Chinese scholars Yu and Wang [47], based on their research on the implicit acceptance of socialist core values among Chinese university students, inferred that students generally endorse these values but have not yet internalized them as their own. This may explain why individuals at this stage accept values cognitively but find it challenging to follow them behaviorally. Moreover, this study not only outlines the cognitive and behavioral characteristics of this stage but also emphasizes that these values are of low priority within the individual’s values system. Schwartz’s [48] theory of values posits that the likelihood of a behavior occurring depends on the relative priority given to relevant, competing values. Therefore, the low prioritization of values and the associated difficulty in behavioral adherence during this stage align with the perspectives in Schwartz’s [48] theory of values.

After individuals accept values, they enter the practice attempt stage, where they begin to try to align their behavior with these values. This stage is characterized by difficulties in practicing the values, external motivation driving their actions, and fear of practicing the values. Similar stages or types of externally motivated action are described in Kelman’s [12] three-stage theory, particularly in the compliance and identification stages, as well as in the introjection stage of Deci et al.’s [13] SDT. Although individuals in this stage attempt to behave in ways consistent with their values under the influence of external motivation, they have not yet fully internalized these values. This is the similarity between this study and the previous internalization theory. Moreover, this study highlights that during the practice attempt stage, individuals often find it difficult to practice values and may experience emotional fear when trying to do so. The gap between intention and behavior is sometimes referred to as the “intention-behavior gap” [49], representing a “black box” of processes. At this stage, even though individuals have accepted the values and begun attempting to practice them, unforeseen obstacles can lead to discrepancies between their intentions and actions [50]. The experience of difficulty and fear in practicing values suggests that there is a gap between behavior and intention, indicating the need for further internalization of the values to narrow this gap.

After individuals attempt to practice values, their acceptance of these values reaches its peak, leading to the voluntary practice of values, marking the integration stage. This stage is characterized by holding and identifying with values, striving to practice values, values guiding behavior, increased initiative in following values driven by internal motivation, enjoying a life aligned with these values, and the values becoming deeply intertwined with life, with high importance and priority. This stage shares similarities with the internalization of the mediational link in Vygotsky’s theory of internalization [10], the internalization stage in Kelman’s [12] three-stage theory, and the integration stage in Deci et al.’s [13] SDT, all of which indicate that behavior consistent with values after internalization is driven by internal motivation. Additionally, this study finds that, in the integration stage, individuals hold and identify with the values. Previous scholars’ definitions of values also reflect that internalized values are cognitively expressed. For example, Rohan [51] views values as beliefs about ideals and goals, representing a stable, high-level cognitive structure that generates meaning. Also, when individuals internalize values, they enjoy living in accordance with these values. Individuals experience positive emotions toward things that align with their values and negative emotions toward things that do not. Empirical studies on emotional identification with values also show that individuals feel positive emotions, such as “satisfaction”, “approval”, and “liking”, toward values they identify with [52]. Moreover, as the degree of values internalization increases, the values become closely related to the individual’s life, and their importance and priority also increase. Yue et al. [53] also found that at the implicit processing level, values considered important are internalized into the self-concept, and the extent to which important values are internalized or excluded from the self-concept is related to their ranking in the individual’s value hierarchy. This finding is consistent with this study, indicating that when values are highly internalized, their closeness to the self and their importance/priority also increase.

**Table 5:** Characteristics of the four stages of values internalization

	Neglect-resistance stage	Understanding stage	Trial action stage	Integration stage
Cognition	Rejecting value information	Initial understanding of values (superficial understanding)		Values endorsement; adhering to values
Behavior	Behavior inconsistent with values	Acceptance of values but difficulty in adhering to them in behavior	Difficulty in practicing values	Efforts to practice values; Behavior guided by values
Motivation			External motivation drives individuals’ behaviors	Increased proactivity in following values; Internal motivation
Emotion	Negative emotions towards values		Fear of living out values	Enjoyment of a value-aligned life
Self-relevance, importance, and priority	Low relevance of values to life; Low importance of values	Low priority of values		Values are related to self; High Importance and Priority

### Motivations for the internalization of values

Each factor in personal development influences the shaping of values [54]. This study categorizes the motivations behind values internalization into internal and external causes. Previous research has identified several factors influencing value formation, such as the transmission of family values [55], genetics [56–58], brain structure [59], life process [54, 60, 61], significant life events [54], social networks [54], cultural differences [62–64], mass media [65, 66], as well as race, ethnicity, gender, social class, education, occupation, family characteristics, religion, economic and political systems [61, 67, 68]. The motivations for value internalization identified in this study align with the factors found in previous research, such as life course (changes in cognitive levels, age, and identity transformation), significant life events, social networks (peer behavior, others’ approval, maintaining relationships with others), media influence, and education from schools and families. In addition, this study identifies other motivations, including achieving positive outcomes and avoiding negative consequences, pursuing achievement, avoiding negative emotions, and gaining positive emotional experiences. Among these, achieving positive outcomes and avoiding negative consequences are the most prominent internal causes identified in this study. Heckhausen’s expectancy-value model also emphasizes that the consequences of behavior are motivating factors, and motivation primarily depends on the value individuals assign to behavioral outcomes [69]. Pursuing achievement is the third most significant internal cause. Theories such as expectancy-value theory, attribution theory, and Bandura’s self-efficacy theory also stress the importance of individuals’ expectations for success [70]. Achievement motivation helps individuals exhibit better behavior; for example, studies have found that individuals with high achievement motivation perform better on simple arithmetic problems and learning tasks than those with low achievement motivation [71]. In terms of emotions, individuals tend to focus on avoiding negative emotional experiences and gaining positive emotional experiences. In the theories of motivation and volition, Kuhl [72] uses emotion control strategies to explain persistence in the face of distractions and other opportunities [70]. In other words, emotional control can also influence whether individuals accept external viewpoints. Therefore, in addition to identifying the same influencing factors as previous research, this study also finds that behavioral outcomes, achievement motivation, and emotion control impact individuals’ value internalization.

### Strengths and limitations

This study, by analyzing interview data, reveals the process, motivations, and characteristics of values internalization, offering several advantages. To begin with, it is the first to categorize the process of values internalization from a subjective perspective. Previous theories have typically approached values internalization from an external observational viewpoint. However, third-person perspectives are limited in directly observing individuals’ conscious experiences [73], whereas first-person perspectives allow for the study of subjective experiences [74]. Therefore, by using interviews to gather information on values internalization from a subjective perspective, this study complements and enriches existing third-person perspective research on values internalization. A second valuable point to mention in this study is that the sample demonstrates strong diversity. A stratified sampling strategy, a type of purposive sampling, was employed to select cases for analysis that could provide rich information [75]. The interviewees in this study come from various occupational groups (including company employees, middle and university teachers, students, and retirees), age groups (ranging from 19 to 58 years old), and educational backgrounds (from high school to master’s degree). This ensures that diversity is incorporated into the sample, maximizing the selection of participants who can provide the most valuable information. Lastly, this study identifies the characteristics of the stages of values internalization at different levels, providing more comprehensive and referable indicators for future measurements of values internalization. Existing empirical studies on values internalization often measure the internalization level using single indicators, which vary across studies. For example, Ryan and Connell [76] and Roth et al. [77] used motivational indicators; Todaro et al. [78] chose behavioral indicators; Kell and Motowidlo [79] measured from a cognitive perspective. Measuring values internalization from a single perspective may fail to accurately and effectively reflect whether individuals hold certain values and may overlook the contributions of other indicators to the measurement of values internalization, making it difficult to comprehensively capture individuals’ actual internalization levels. The indicators identified in this study include cognition, behavior, motivation, emotion, self-relevance, importance, and priority, forming a multidimensional model that helps improve the validity of value internalization measurements.

This study also has several limitations. First, regarding the research participants, all the interviewees in this study are from mainland China, and values can differ significantly across cultures [80] Expanding the research on the process of value internalization to other countries, regions, or subcultural groups (such as ethnic minorities) is necessary to further validate the accuracy of the value internalization process model. Second, while interviewing is a powerful way of getting insights into interviewees’ perceptions, it is susceptible to subconscious biases and potential inconsistencies [81]. Participants might overemphasize certain events or interpret past experiences in light of current beliefs. To address these limitations, future research could adopt longitudinal designs that follow individuals over time, thereby capturing the dynamic process of value internalization as it unfolds in real life. In addition, neuroscience data collection methods do not require participants to provide verbal responses, meaning that the measurement itself does not interfere with the cognitive processes being studied. Additionally, neuroimaging measurements are based on biological signals of processes, allowing for continuous recording. This temporal dimension is indispensable for process research. Moreover, neuroimaging can better avoid social desirability bias and is not affected by recall distortion [82]. Together, longitudinal and neuroscientific approaches may help overcome the limitations associated with interview data and contribute to a more comprehensive understanding of how values are internalized.

## Conclusion

Based on the overall discussion of the values internalization process, the main conclusions are as follows: (a) The values internalization process includes four stages: neglect-resistance stage, understanding stage, trial action stage, and integration stage. Notably, our findings highlight the previously understudied neglect-resistance stage, where initial resistance and value dismissal may constitute a critical prerequisite for subsequent internalization processes. (b) The various stages of values internalization exhibit distinct characteristics in cognition, behavior, motivation, emotion, self-relevance, importance, and prioritization. These distinctions offer valuable insights for future assessments and measurements of values internalization. (c) The primary internal factors influencing values internalization are the pursuit of positive outcomes and the avoidance of negative consequences, while the main external factors include experiencing significant life events and the behavior of peers.

## Data Availability

The raw data supporting the conclusions of this article will be made available by the authors, without undue reservation.
